# Subliminal perception of others’ physical pain induces personal distress rather than empathic concern

**DOI:** 10.1186/s40359-023-01310-3

**Published:** 2023-09-15

**Authors:** Juan Song, Zijing Zhao, Zhibin Jiao, Yao Peng, Mingyuan Chu

**Affiliations:** 1 Faculty of Psychology, Tianjin Normal University, Tianjin, China; 2 Dongguan Nancheng Middle School, Dongguan, China; 3 School of Psychology, University of Aberdeen, Aberdeen, UK

**Keywords:** Subliminal priming, Pain perception, Emotional and behavioural response, Threat value of pain hypothesis, Empathy-altruism hypothesis

## Abstract

**Background:**

What is our immediate reaction when we witness someone experiencing pain? The empathy-altruism hypothesis predicts that observers would display empathy and a tendency to approach the person in pain. Alternatively, the threat value of pain hypothesis (TVPH) argues that others' pain serves as a signal of threat and should induce observers’ avoidance response.

**Methods:**

To examine these two hypotheses, three experiments were conducted. The experiments aimed to investigate the impact of subliminal exposure to others' physical pain on participants' emotional and behavioural responses.

**Results:**

The results revealed that subliminal pain priming resulted in faster response and attentional bias to fearful faces compared to sad faces (Experiment 1), faster reaction times in recognizing fear-related words compared to anger-related words during a lexical decision task (Experiment 2), and faster avoidance responses towards anger-related words, as opposed to approaching responses towards positive words (Experiment 3).

**Conclusions:**

The consistent findings across all experiments revealed that subliminal perception of pain scenes elicited fear emotion and immediate avoidance responses. Therefore, the outcomes of our study provide supportive evidence for the TVPH.

**Supplementary Information:**

The online version contains supplementary material available at 10.1186/s40359-023-01310-3.

## Background

Pain serves as a warning signal, alerting us to potential or existing physical harm resulting from exposure to harmful stimuli [1]. When we encounter something excessively hot, cold, or sharp, the sensation of pain prompts us to withdraw to minimize further injury to our bodies. Pain can be personally experienced or observed in others. The pain experienced by others not only evokes unpleasant feelings in observers but also serves as a reminder of the potential dangers present in the environment.

In our daily lives, we frequently come across situations where we witness others experiencing pain, such as car accidents, finger cuts, burns, or falls. According to Grynberg and Konrath [2], when we observe others in pain, we often undergo two distinct emotional responses: empathic concern and personal distress. Empathic concern tends to lead to caring and altruistic behaviours towards others, while personal distress is associated with a motivation to withdraw and avoid potential external threats. The empathic concern and personal distress responses proposed by Grynberg and Konrath [2] are also align with the empathy-altruism hypothesis [3] and the threat value of pain hypothesis (TVPH) [1, 4, 5]. The empathy-altruism hypothesis proposes that witnessing others' pain triggers empathic concern in observers, prompting them to approach and provide support. On the other hand, the TVPH suggests that perceiving others' pain does not automatically activate empathic responses, but rather activates the perceiver's threat-detection system, inducing avoidance behaviours.

The goal of the present study was to assess these seemingly contradictory predictions by examining the emotional and behavioral responses elicited by subliminal perception of other people's physical pain. The empathy-altruism hypothesis would predict approach-oriented emotional and behavioural responses, while the TVPH would predict avoidance-oriented emotional and behavioural responses.

### Empathy-altruism hypothesis

The empathy-altruism hypothesis suggests that witnessing others in need can lead to empathic concern and motivate people to help [3, 6]. Studies by Batson et al. [7] and Van Lange [8] provided evidence for this hypothesis. In Batson et al.'s study, participants listened to a recording of a protagonist who had tragically lost her parents in a car accident. It was found that those who listened to the recording from the protagonist's perspective (high empathy condition) were more willing to help compared to those listened from an objective perspective (low empathy condition). Similarly, Van Lange's study found that participants with higher empathy levels towards the protagonist showed stronger altruistic motivation. These studies consistently support the role of empathy in promoting altruistic behaviors.

As studies mentioned previously have primarily focused on social pain experiences, it is unclear if witnessing physical pain (e.g., cutting the finger with a knife) elicits similar empathic and altruistic behaviors compared to social pain. Batson et al. [7] suggested that individuals may experience personal distress when observing physical pain, while empathic concern is more likely to be experienced in social pain situations. In a study by Fabi et al. [9], participants were presented with scenarios involving social, physical, or no pain. They rated their empathic concern and personal distress levels while performing a response task based on high and low tones. The results showed stronger empathic concern in the social pain condition and stronger personal distress in the physical pain condition. However, there were no differences in response tendencies (approaching vs. avoidance) between the two conditions, possibly due to the timing of the tones, which occurred 1000 ms after the image presentation, potentially diminishing the empathic concern or personal distress response tendencies by that point.

### Threat Value of Pain Hypothesis (TVPH)

The threat value of pain hypothesis (TVPH) suggests that observing others in pain is perceived as a potential threat. Rather than triggering empathic or approaching responses, it activates the observer's threat detection system, leading to an avoidance response [1, 4, 5]. In Yamada and Decety [5], participants were first presented with subliminal priming stimuli consisting of positive words (e.g., honest), negative words (e.g., rude), or scrambled words as a neutral condition. They were then shown images of painful and happy facial expressions and were asked to determine whether the face expressed pain or not. Participants exhibited greater sensitivity to pain faces when primed with negative words, but no differences were found under positive or neutral word priming conditions. This finding suggests a link between the perception of words with higher threat value and the detection of pain.

In a study by Ibáñez et al. [4], participants were primed with either their own faces or faces of others, assuming that others' faces represent a stronger threat. They were then presented with neutral or painful semantic stimuli (Experiment 1) or neutral or painful pictures (Experiment 2). Participants had to judge whether the target stimuli were painful or neutral while their ERPs data were recorded. The study found that participants responded faster and showed greater cortical activity when seeing painful stimuli after being primed with others' faces, indicating a higher perceived threat value. This suggests that a higher threat value leads to quicker and stronger responses to pain stimuli.

Additionally, Morrison et al. [10] found that observing others' physical pain leads to avoidance responses. Participants watched a brief video of a hand being pricked with a needle (physical pain condition) or touching cotton (non-painful condition). After a 500 ms interval, they had to press (approach) or release (avoidance) buttons in response to target shapes presented in different colours. The results revealed that participants had faster release (avoidance) responses and slower press (approach) responses in the physical pain condition compared to the no-pain condition. These findings suggest that the perception of others' physical pain triggers avoidance responses in observers. The results showed that participants exhibited faster release (avoidance) responses and slower press (approach) responses in the physical pain condition compared to the no-pain condition. These findings indicate that the perception of physical pain in others promotes avoidance responses in observers.

Taken together, these studies showed that after being primed by high threat value stimuli, participants showed stronger responses to pain stimuli. Thus, there was a clear association between pain and stimuli with high threat value. However, none of these studies directly tested the effect of pain perception on participants’ emotional and behavioural responses.

### The Present Study

The goal of the present study is to directly evaluate the empathy-altruism hypothesis and the TVPH by examining the effect of pain scene priming on participants' subsequent emotional and behavioral responses. Three experiments were conducted to examine the emotional and behavioral responses after perceiving the pain scene. In all three experiments, participants were presented with subliminal primes of pain and no-pain scenes. They then completed a dot-probe task with sad and fearful faces as target stimuli in Experiment 1, a lexical decision task with fear-related, anger-related, and neutral words as target stimuli in Experiment 2, and an approach/distancing decision task with fear-related, anger-related, and positive words as target stimuli in Experiment 3.

In this study, subliminal priming was utilized to induce subliminal perception, which refers to the phenomenon where a stimulus influences cognition, emotion, action, learning, or memory without conscious awareness [11]. Subliminal perception operates below the subjective threshold and is less susceptible to individual experiences and attitudes, thus minimizing the potential for social desirability bias [12]. Subliminal perception holds particular significance for individual survival, as it can trigger rapid primitive biological responses such as escape or defense [13, 14]. Moreover, in line with the findings of previous subliminal priming studies, the congruency effect suggests that participants are expected to exhibit faster and more accurate responses to target stimuli that are congruent with the subliminal prime, compared to those that are incongruent [15].

There is evidence to suggest that the motivational dimension of emotions may vary depending on the task involved [16]. Previous research indicates that the effects of emotions on approach-avoidance tendencies are more likely to be observed when participants explicitly focus on the approach/avoidance dimension, such as in an approach/distancing decision task, as opposed to when they do not focus on this dimension, such as in a lexical decision task [17]. Therefore, we conducted three experiments with a dot probe task, a lexical decision task and an approach/distancing decision task to investigate the consistency of the effect of subliminal pain perception on participants' approach-avoidance tendencies across paradigms. If the effect was robust, the TVPH would predict faster and more accurate response to stimuli associated with avoidance tendencies compared to stimuli associated with approach tendencies. In contrast, the empathy-altruism hypothesis would predict opposite outcomes.

### Experiment 1

Experiment 1 investigated the effect of subliminal pain scene priming on participants' emotional response using a dot probe task [18]. Pairs of emotional faces (sad versus neutral or fearful versus neutral) were briefly presented simultaneously, followed by a prompt (white dot) appearing on the target emotional face side (congruent trials) or the neutral face side (incongruent trials). Participants indicated the prompt's location, allowing the calculation of attentional bias scores. This task assessed selective attention towards emotional stimuli [19] and examined whether attentional bias resulted from accelerated attentional orientation or difficulty in disengaging attention. Furthermore, the motivational dimension of emotion suggests that different emotions have varying inclinations towards approach or avoidance tendencies [16, 20–22]. For example, sadness and anger are associated with an approach tendency [23–26], while fear is linked to avoidance [27]. Based on the TVPH, participants primed with a pain scene were expected to respond faster and show attentional bias towards fearful faces, reflecting potential danger and avoidance tendencies [28]. Conversely, according to the empathy-altruism hypothesis, faster responses and attentional bias towards sad faces would be expected, indicating a strong desire for help and an approach tendency [29].

## Method

### Participants

Given the unknown effect size, we decided to set a medium effect size (f) of 0.25. A sensitivity analysis conducted in G-power suggested that a minimum of 16 participants would be required to achieve a power of 80% with a significance level (α) of 0.05. Twenty-five students from Tianjin Normal University (19 females and 6 males) with an average age of 20.04 years (*SD* = 1.49; *range* = 18–24 years) participated in Experiment 1. All participants had normal or corrected-to-normal vision and received compensation of 15 RMB for their participation. Informed consent was obtained from all participants, and they were free to withdraw from the study at any time. The study was approved by the University Ethics Committee at Tianjin Normal University.

### Design

A 2 (priming type: pain, no pain) × 2 (negative emotion type: sadness, fear) × 2 (the site of probe dot: consistent, inconsistent) within-subject experimental design was used. Response time (RT) and attentional bias indexes were the dependent variables. The attentional bias indexes include attentional bias score (RT of inconsistent condition—RT of consistent condition), attentional orientation acceleration score (RT of neutral–neutral condition—RT of negative-neutral condition) and disengagement difficulty score (RT of neutral-negative condition—RT of neutral–neutral condition) [30].

The attentional bias score measured the attentional results of individuals, that was, whether they showed attentional bias to negative stimuli. The attentional orientation acceleration score and disengagement difficulty score served as explanatory factors for the observed attentional bias. These scores help elucidate that participant either demonstrated quicker attentional capture towards a stimulus or experienced difficulties in disengaging their attention from a stimulus. To establish these scores, neutral faces were used as a baseline for calculating the acceleration and disengagement difficulty scores.

### Materials

#### Priming stimuli

**Fig. 1:**
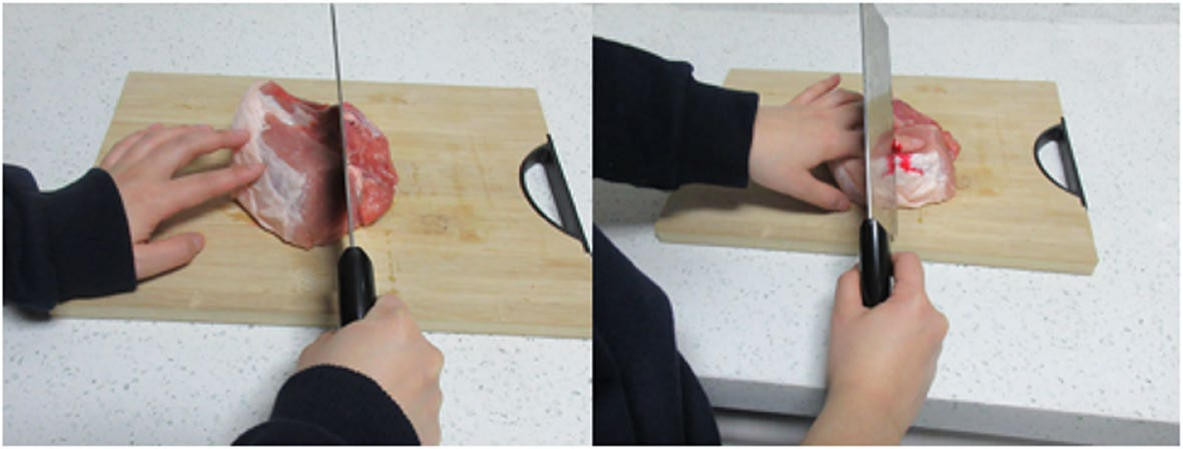
Example of a no pain and a pain scene

Sixteen images of no pain scenes and sixteen pain scenes adopted from Lei [31]were selected. Four no pain images and four pain images were used in the practice trials. All images were standardized to have identical color contrast, size, and brightness. An illustrative example of these images can be seen in Fig. 1.

#### Masking stimuli

**Fig. 2:**
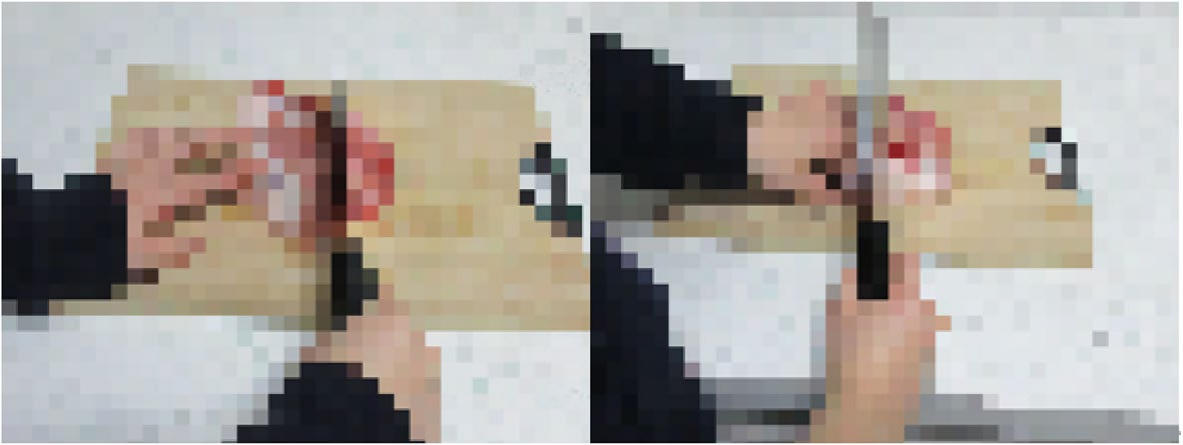
Example masked images of a no pain and a pain scene

To ensure the effectiveness of subliminal priming, masking pictures were employed in the study. Prior research has demonstrated that the presence of post-masking images is essential to secure the priming effect [32]. Adobe Photoshop software was utilized to uniformly mask the priming pictures, with dimensions of 7cm × 9cm and a resolution of 100 pixels/inch (see Fig. 2 for an example).

#### Emotional faces

**Fig. 3:**
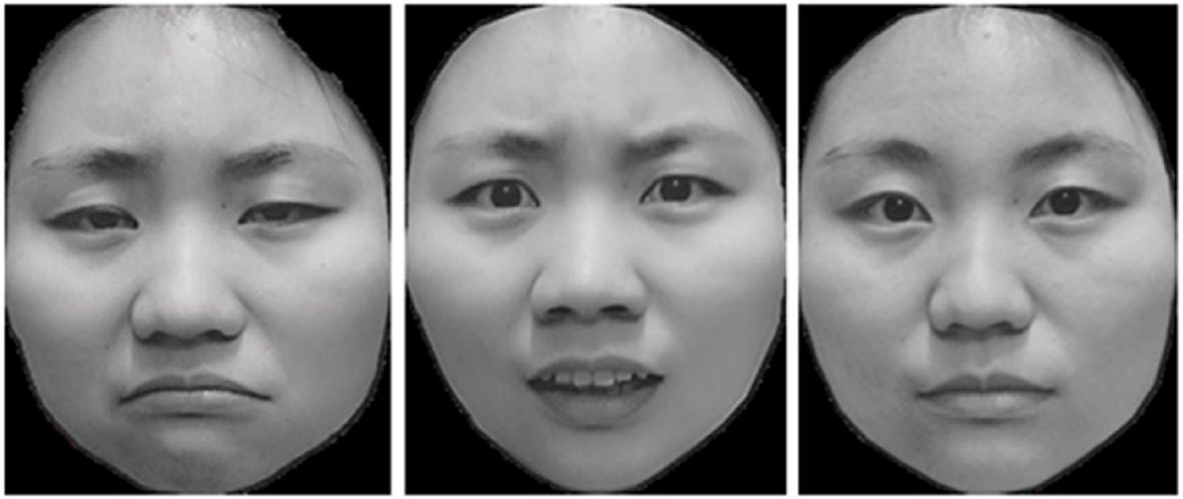
Example of emotional faces from a female model (From left to right: sadness, fear, neutral)

The target stimuli were sad, fearful, and neutral faces adopted from Peng [33]. A total of 12 models (6 males) were screened, with three emotional facial pictures (sad, fearful and neutral) from each model (see Fig. 3 for an example). These emotional facial pictures of each model were used to generate three emotional face pairs (sad-neutral, fearful-neutral, and neutral–neutral).

### Procedure

The participants were tested individually in a quiet room, seated in front of a computer. After giving informed consent, the participant first completed 12 practice trials, followed by 288 experimental trials. For the experimental trials, 12 pain and 12 no pain priming pictures were used. Each priming picture was repeated six times in both the consistent dot-probe condition and the inconsistent dot-probe condition. For the emotional face pairs, there were 36 pairs (sad-neutral, fearful-neutral, neutral–neutral) from 12 models. The site of each emotional face was counterbalanced, which led to 72 emotional face pairs. These pairs were then combined with the priming conditions (pain, no pain) and the two dot-probe conditions (consistent, inconsistent), resulting in a total of 288 experimental trials.

**Fig. 4:**
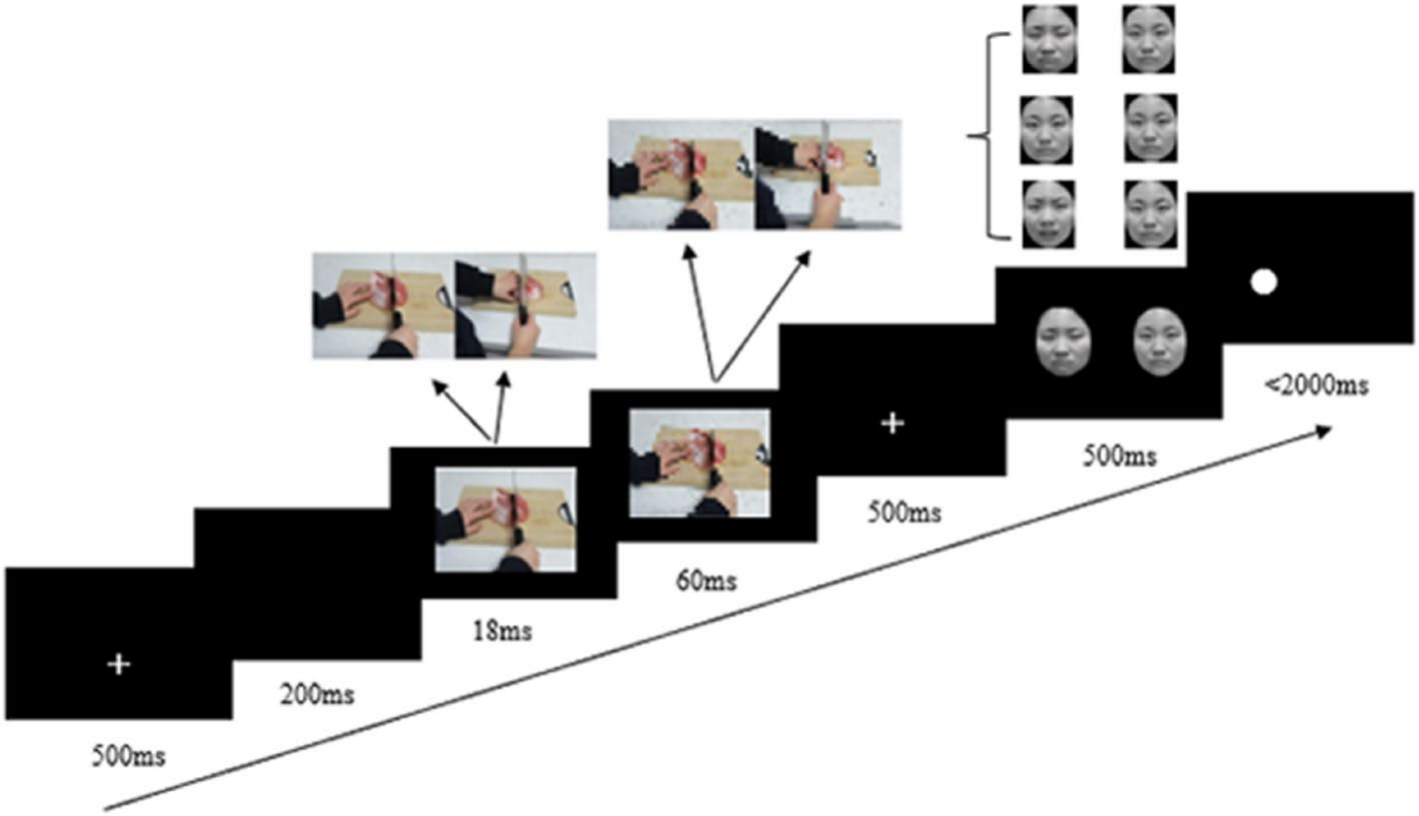
Illustration of the procedure in Experiment 1

Each trial followed the steps outlined in Fig. 4: (1) A white fixation cross appeared at the center of the screen for 500ms; (2) A blank screen was presented for 200ms; (3) The priming picture depicting either a pain scene or a no pain scene was displayed for 18ms. Previous studies have indicated that a presentation time ranging from 12 to 20ms can reliably induce a subliminal priming effect [4, 34–36]. In our study, based on the results of a pilot study, we used a presentation time of 18ms; (4) Following the priming picture, a masked picture was presented for 60ms [35]; (5) A white fixation cross reappeared on the screen for 500ms; (6) The emotional face pair (sad-neutral faces, fearful-neutral faces, neutral–neutral faces) were then displayed on the screen with each face on one side (left or right) of the screen for 500ms; (7) After the faces disappeared, a white dot appeared either on the same side as the negative emotional face (consistent) or on the same side as the neutral face (inconsistent). The dot was positioned in the center of a face. Participants were instructed to judge the position of the dot as quickly as possible by pressing the "F" key for left or the "J" key for right. Participants had maximally 2000 ms to respond. If no response was recorded within 2000 ms, the dot would disappear and the next trial would commence. The inter-trial interval was 200 ms.

After the experiment, we checked the effectiveness of the subliminal priming by asking participants whether they noticed the flash screen before the masked image appeared and, if so, whether they were able to identify the content of the flash screen. None of the participants reported perceiving the flash screen. This indicates that the subliminal priming was successful. The entire experiment lasted approximately 15 min.

### Data analysis

Before conducting data analysis, data were excluded according to the following criteria: (1) participants with an error rate higher than 20%; (2) RTs below 200 ms; (3) RTs exceeded 3 standard deviations the mean RT of a participant; (4) Trials with no responses or incorrect response. Based on these criteria, 2.31% of the data were excluded from the analyses. For all experiments and analyses, a significance level of *α* = 0.05 was adopted. A level of marginal significance was considered for 0.05 < *α* < 0.1. In cases where the assumption of sphericity was violated, the Greenhouse–Geisser correction was applied. Post hoc corrections were carried out using the LSD method to account for multiple comparisons.

## Results

### RTs

**Fig. 5:**
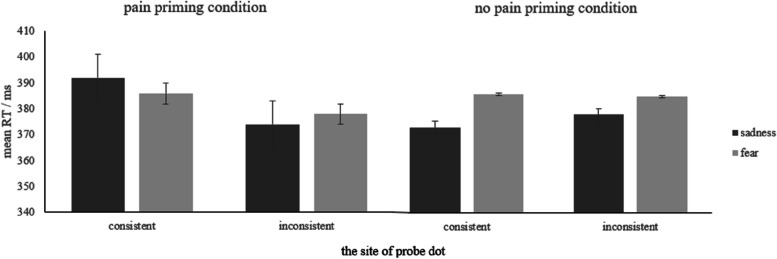
Mean RT of each condition. Error bars represent standard errors

A repeated-measure ANOVA was conducted with the priming type (pain, no pain), negative emotion type (sadness, fear) and the site of probe dot (consistent, inconsistent) as the independent variables, and the mean RT of the correct responses as the dependent variable. Descriptive statistics are presented in Fig. 5, and full results can be found in S1 Table 1 in the Supplemental Material. The results revealed a main effect of negative emotion type (*F* (1, 24) = 4.94, *p* = 0.036, *η*_*p*_^*2*^ = 0.17) and a main effect of the site of the probe dot (*F* (1, 24) = 12.04, *p* = 0.002, *η*_*p*_^*2*^ = 0.33). Furthermore, significant two-way interactions were observed between the priming type and negative emotion type (*F* (1, 24) = 14.77, *p* = 0.001, *η*_*p*_^*2*^ = 0.38), as well as between the priming type and the site of the probe dot (*F* (1, 24) = 12.40, *p* = 0.002, *η*_*p*_^*2*^ = 0.34). Lastly, there was a significant three-way interaction among the priming type, negative emotion type and the site of probe dot (*F* (1, 24) = 5.71, *p* = 0.025, *η*_*p*_^*2*^ = 0.19).

To further examine the three-way interaction, separate 2 (negative emotion type: sadness, fear) × 2 (the site of probe dot: consistent, inconsistent) ANOVAs were conducted for each priming type (pain, no pain). Under the pain priming condition (see S1 Table 2 for full results), the main effect of the site of probe dot (*F* (1, 24) = 21.45, *p* < 0.001, *η*_*p*_^*2*^ = 0.47) and the interaction between negative emotion type and the site of probe dot (*F* (1, 24) = 5.37, *p* = 0.029, *η*_*p*_^*2*^ = 0.18) were significant. The RT of sadness was marginally longer than fear (*p* = 0.089) when the site of the probe dot was consistent, whereas there was no significant RT difference between sadness and fear faces when the site of the probe dot was inconsistent. Under the no pain priming condition (see S1 Table 3 for full results), there was only a significant main effect of the negative emotion type (*F* (1, 24) = 25.48, *p* < 0.001, *η*_*p*_^*2*^ = 0.52). Participants responded faster to sadness than fear faces (*p* < 0.001). However, there were no significant effects related to the site of the probe dot. Thus, the three-way interaction can be explained as follows: when primed by pain scenes, participants responded faster to fear faces when the site of the probe dot was consistent, but equally fast to both sadness and fear faces when the site of the probe dot was inconsistent. When primed by no-pain scenes, participants responded faster to sadness faces regardless of the site of the probe dot.

### Indices of attentional bias

**Fig. 6:**
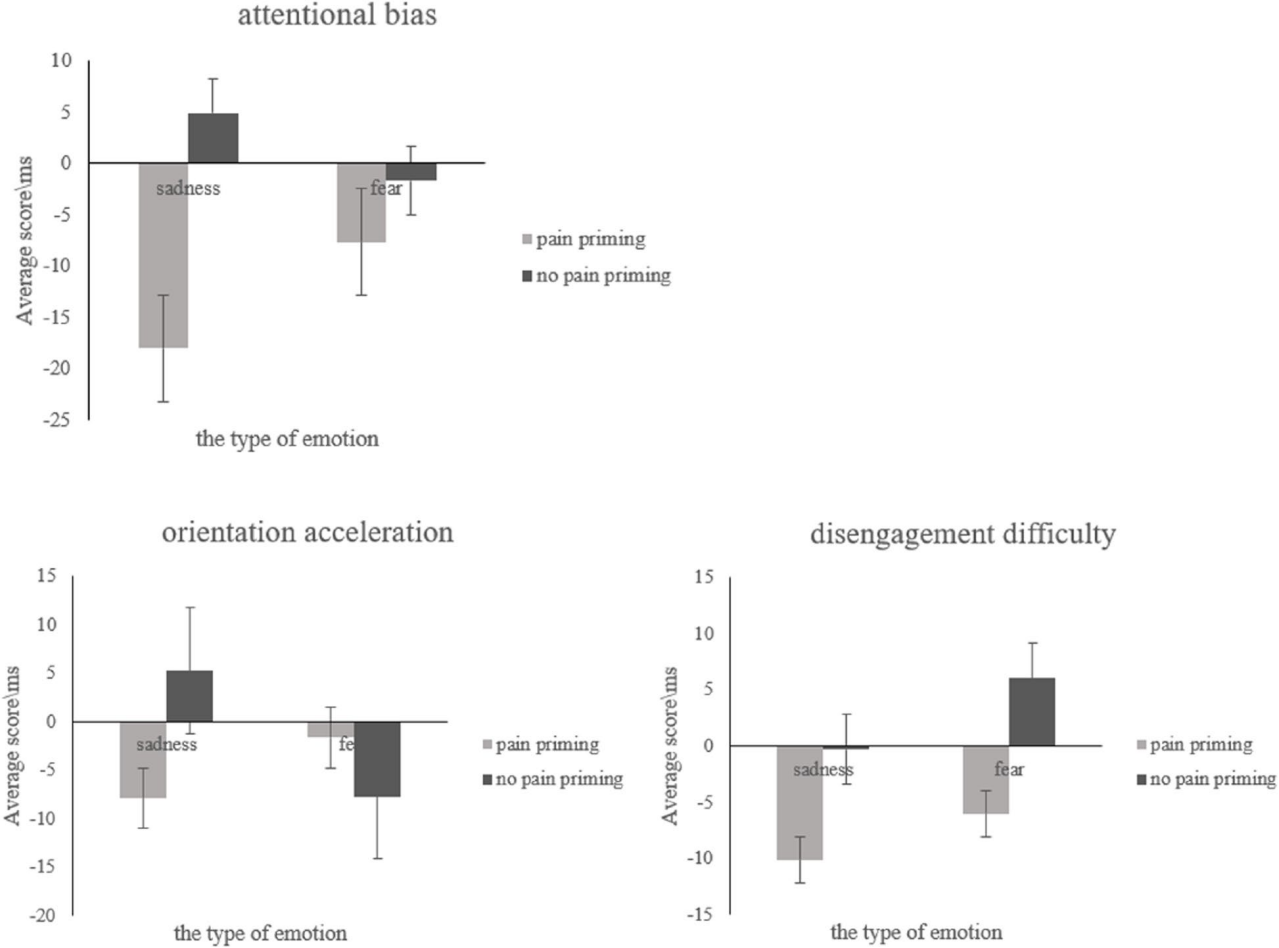
Mean attentional bias indices of each condition. Error bars represent standard errors

Three repeated-measure ANOVAs were conducted with the priming type and negative emotion type being independent variables, and the three indices of attentional bias being dependent variables. Figure 6 presents the descriptive statistics of different attentional bias indices.

#### The Attentional bias score (see S1 Table 4 for full results)

There was a significant main effect of priming type (*F* (1, 24) = 12.40, *p* = 0.002, *η*_*p*_^2^ = 0.34). The interaction between the priming type and negative emotion type was significant (*F* (1, 24) = 5.71, *p* = 0.025, *η*_*p*_^2^ = 0.19). Under pain priming, individuals had a higher attentional bias score for fearful faces than for sad faces (*p* = 0.029), whereas under no pain priming, the attentional bias score did not differ between the two negative emotion types.

#### The attentional orientation acceleration score (see S1 Table 5 for full results)

There was a significant interaction between the priming type and the negative emotion type (*F* (1, 24) = 20.49, *p* < 0.001, *η*_*p*_^2^ = 0.46). Under pain priming, the attentional orientation acceleration score for fearful faces was marginally higher than for sad faces (*p* = 0.089), whereas under no pain priming, the score for sad faces was higher than for fearful faces (*p* = 0.002).

#### The attentional disengagement difficulty score (see S1 Table 6 for full results)

The main effects of the priming type (*F* (1, 24) = 8.63, *p* = 0.007, *η*_*p*_^2^ = 0.26) and the negative emotion type (*F* (1, 24) = 4.53, *p* = 0.044, *η*_*p*_^2^ = 0.16) were both significant. The attentional disengagement difficulty score was lower under pain priming than under no pain priming. The score for fearful faces was higher for sad faces, which showed that individuals’ attention was harder to disengage from fearful faces than from sad faces.

## Discussion

The results from the RT analyses demonstrated that when participants were subjected to pain priming, they responded faster to fearful faces than to sad faces when the dot appeared on the site of the negative emotional faces. In contrast, under the no-pain priming condition, participants responded to fearful and sad faces equally fast regardless of the site of the dot.

In terms of attentional bias, the results revealed that participants showed attentional bias toward fearful faces under the pain prime but not under the no-pain prime. This bias was due to the faster attention engagement to fearful faces than to sad faces under pain prime but not under the no-pain prime.

Taken together, these results supported the TVPH, as the subliminal pain perception led to faster response to and stronger attentional bias towards fear emotion associated with avoidance tendencies than sadness emotion associated with approaching tendencies.

### Experiment 2

Experiment 2 aimed to test the empathy-altruism hypothesis and the TVPH with a lexical decision task. Participants were first presented with identical subliminal pain scenes used in Experiment 1. They were then shown a two-character Chinese word (i.e., fearful/fear-related, angry/anger-related, neutral, and false words) on the screen and asked to determine whether the word was true or false. Unlike Experiment 1 in which fear and sadness facial images were used and theses images were more directly linked to emotional responses, emotional words used in Experiment 2 required inferential analysis [29]. If the TVPH remained applicable, participants should respond more quickly and accurately to fear-related words than to anger-related words after being primed by the pain scene, as fear-related words were related to avoidance tendency. The empathy-altruism hypothesis would predict the opposite, as anger-related words were related to approach tendency [16].

There were two subtle differences between Experiment 1 and Experiment 2 regarding the stimulus materials. First, we used anger-related words rather than sadness-related words as the target stimulus. This is because previous research has consistently shown that anger-related words can elicit approach responses in a lexical decision task [16]. Second, the priming scene was not masked. This was because previous research has shown that masking the priming picture would reduce the priming effect on the target words in a lexical decision task [37]. Thus, we removed the mask to maximize the priming effect.

## Method

### Participants

A sensitivity analysis conducted in G-power suggested that a minimum of 19 participants would be required to achieve a power of 80% with a medium effect size (f) of 0.25 and a significance level (α) of 0.05. Twenty-five students from Tianjin Normal University (20 females and 5 males) with an average age of 20.00 years (*SD* = 1.12; *range* = 18–23 years) participated in Experiment 2. All participants had normal or corrected-to-normal vision and received compensation of 10 RMB for their participation. Informed consent was obtained from all participants, and they were free to withdraw from the study at any time. The study was approved by the University Ethics Committee at Tianjin Normal University.

### Design

A 2 (priming type: pain, no pain) × 3 (word emotion type: fear-related, anger-related, neutral) within-subject design was used. The mean RT of correct responses and the proportion of correct responses were the dependent variables.

### Materials

#### Priming stimuli

Fifteen images of no pain scenes and fifteen pain scenes adopted from Lei [31] were selected. Three no pain images and three pain images were used in the practice trials. All images were standardized to have identical color contrast, size, and brightness.

#### Target stimuli

Nine fear-related Chinese words (e.g., “窒息” smothering in English), nine anger-related Chinese words (e.g., “荒谬” ridiculous in English), nine neutral Chinese words (e.g., “零星” sporadic in English) and twenty-seven false words (e.g., “格偷” grid-steal in English) were used for the lexical decision task. One true word in each of the three types and three false words were used in the practice trials. All the true words were selected from the Chinese affective words system (CAWS) by Wang [38]. The selected true words were balanced in valence, arousal, familiarity, specificity, word frequency, the total number of strokes and separated emotion dimensions (happiness, sadness, disgust, fear and anger). The first six dimensions were all taken from the CAWS system. In addition, 90 individuals were recruited to evaluate the separated emotion dimension of the selected words (see S2 Table 1).

### Procedures

The participants were tested individually in a quiet room, seated in front of a computer. After giving informed consent, the participant first completed 6 practice trials, followed by 96 experimental trials. For the experimental trials, 12 pain and 12 no pain priming pictures were used. Each priming picture was repeated four times according to word emotion type. For the emotional word, there were four kind words (8 fear-related words, 8 anger-related words, 8 neutral words and 24 false words). The site of each emotional face was counterbalanced, which led to 72 emotional face pairs. Each word was repeated twice according to two kinds of priming pictures, resulting in a total of 288 experimental trials.

**Fig. 7:**
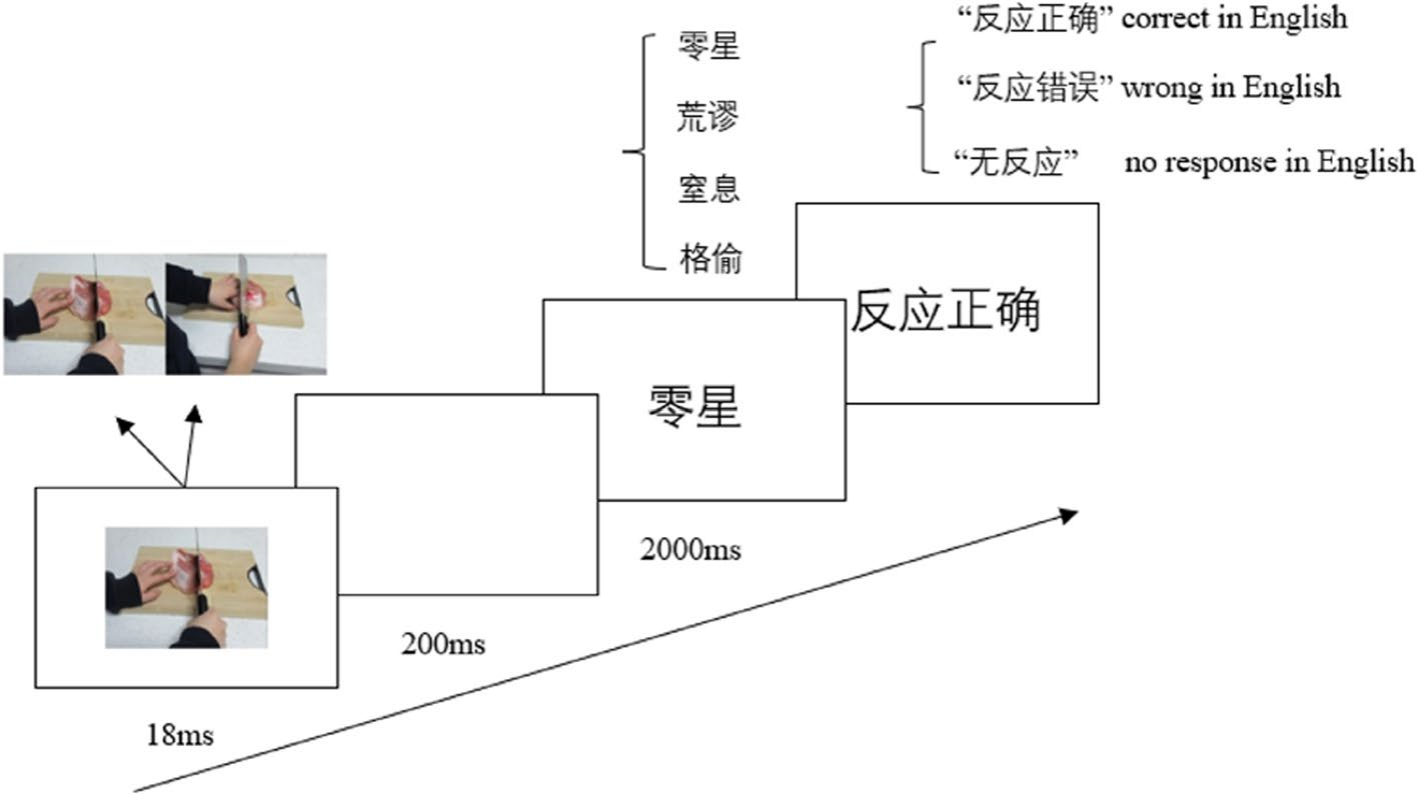
Illustration of the procedure in Experiment 2

Each trial followed the steps outlined in Fig. 7: (1) The priming picture depicting either a pain scene or a no pain scene was displayed for 18ms; (2) A blank screen was shown for 200ms; (3) A two-character Chinese word (fear-related, anger-related, neutral or false) was presented in the centre of the screen. Participants were instructed to judge whether the word was a true word or a false word as quickly as possible by pressing the correspondent keys on the keyboard (“F” and “J” keys). Participants had maximally 2000 ms to respond; (4) A feedback screen (correct, wrong or no response) was displayed for 1500 ms; (5) The inter-trial interval was 1000 ms.

After the experiment, we checked the effectiveness of the subliminal priming by asking participants whether they noticed the flash screen before the word appeared and, if so, whether they were able to identify the content of the flash screen. None of the participants reported perceiving the flash screen. This indicates that the subliminal priming was successful. The entire experiment lasted approximately 10 min.

### Data analysis

Following the exclusion criteria used in Experiment 1, 4.67% of the data was excluded in this experiment.

## Results

### RTs

**Fig. 8:**
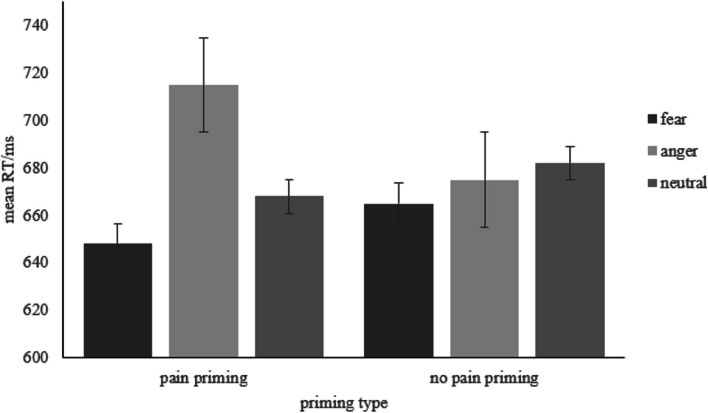
Mean RT of each condition. Error bars represent standard errors

A repeated measure ANOVA was conducted with the priming type (pain, no pain) and word emotion type (fear, anger, neutral) as the independent variables, and the mean RT of correct responses as the dependent variable. Descriptive statistics are presented in Fig. 8, and full results can be found in S2 Table 2 in the Supplemental Material. The results revealed a significant main effect of word emotion type (*F* (2, 48) = 7.62, *p* = 0.003, *η*_*p*_^*2*^ = 0.24). The interaction between priming type and word emotion type was significant (*F* (2, 48) = 3.47, *p* = 0.039, *η*_*p*_^*2*^ = 0.13). Paired-sample t-tests revealed that under the pain priming, the RT for fear words was significantly shorter compared to anger words (*p* = 0.001), and the RT for anger words was significantly longer compared to neutral words (*p* = 0.007). However, under the no pain priming, RTs did not differ between different emotion word types.

### Accuracy

**Fig. 9:**
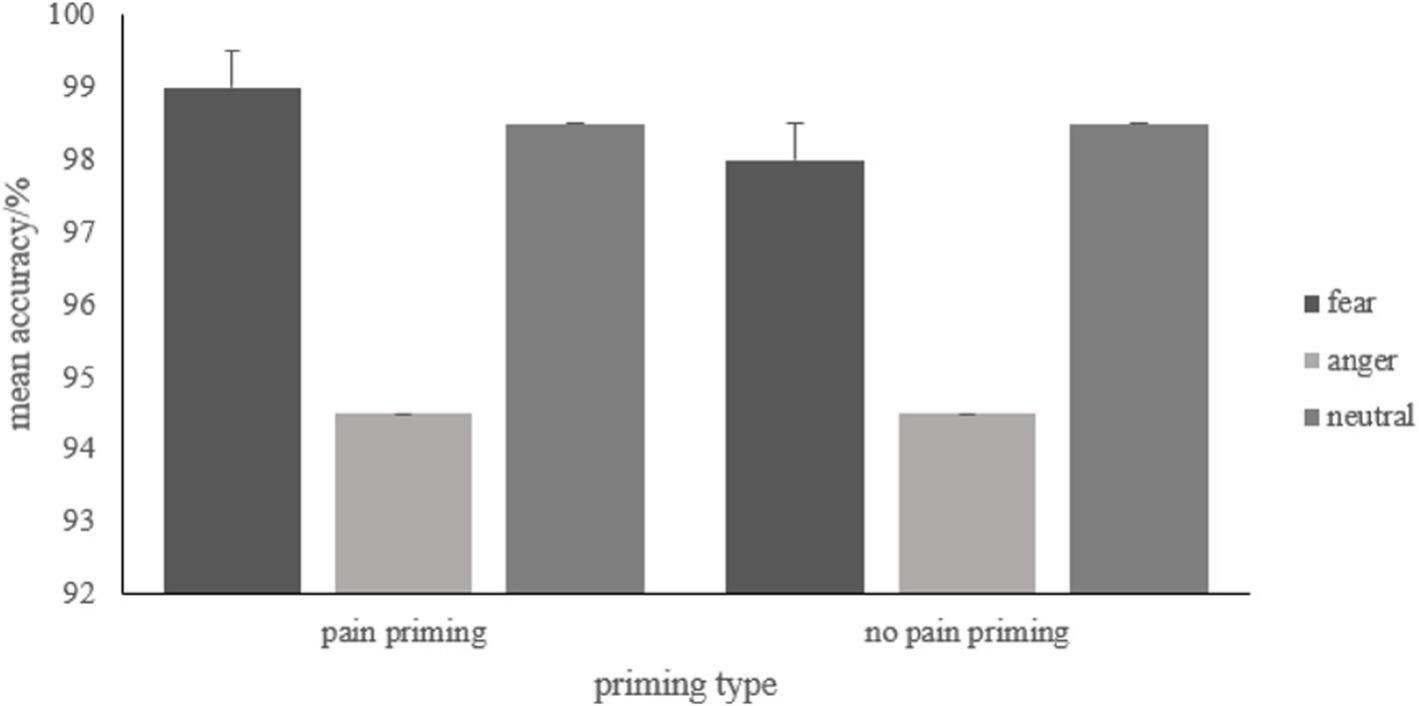
Mean accuracy of each condition. Error bars represent standard errors

A repeated measure ANOVA was conducted with the priming type (pain, no pain) and word emotion type (fear-related, anger-related, neutral) as the independent variables, and the proportion of correct responses as the dependent variable. A correct response to an emotion word is when participants correctly judge an emotion word as a true word. Descriptive statistics are presented in Fig. 9, and full results can be found in S2 Table 3 in the Supplemental Material. The results revealed a main effect of word emotion type (*F* (2, 48) = 7.28, *p* = 0.004, *η*_*p*_^2^ = 0.23). The accuracy for anger-related words was significantly lower than for fear-related words (*p* = 0.007) and for neutral words (*p* = 0.008).

## Discussion

RT results revealed that the RT to fear-related words was quicker than to anger-related words under the pain priming, whereas the RT to the fear-related and anger-related words did not differ under the no-pain priming. This suggests that the pain scenes were more strongly associated with fear emotion than with anger emotion, supporting the TVPH. Accuracy results indicated that participants responded more accurately to fear-related words than to anger-related words regardless of the type of priming. This might be because the recognition of fear holds important evolutionary significance.

### Experiment 3

Experiments 1 and 2 provided compelling evidence that individuals did unconsciously respond to pain scenes with fear emotions, which is associated with avoidance tendency. Experiment 3 aimed to examine the empathy-altruism hypothesis and the TVPH with an approach/distancing decision task (ADDT), where participants were explicitly instructed to perform either an avoidance or an approaching response to emotional words. Participants were first presented with identical subliminal pain scenes used in previous experiments, and then they were presented with a two-character Chinese word (fear-related, anger-related, and positive words) on the screen and were asked to judge whether they wanted to approach or avoid the word. According to the TVPH, participants’ avoidance response to fear and anger-related words should be faster and more accurate than their approach response to positive words, whereas the empathy-altruism hypothesis would predict the opposite. In addition, participants’ general approach/avoidance behaviour tendency was used as a covariable in the analysis, which allowed us to focus solely on participants' approach/avoidance responses to the target stimuli following the subliminal perception of pain scenes.

## Method

### Participants

A sensitivity analysis conducted in G-power suggested that a minimum of 19 participants would be required to achieve a power of 80% with a medium effect size (f) of 0.25 and a significance level (α) of 0.05. Forty-one students from Tianjin Normal University (20 females and 5 males) with an average age of 19.90 years (*SD* = 0.86; *range* = 18–21 years) participated in Experiment 3. All participants had normal or corrected-to-normal vision and received compensation of 10 RMB for their participation. Informed consent was obtained from all participants, and they were free to withdraw from the study at any time. The study was approved by the University Ethics Committee at Tianjin Normal University.

### Design

A 2 (priming type: pain, no pain) × 3 (word emotion type: fear-related, anger-related, positive) within-subject experimental design was used. The mean RT of correct responses and the proportion of correct responses were the dependent variables. Participants’ own approach/avoidance behaviour tendencies were treated as covariables in the analyses.

### Materials

#### Priming stimuli

Ten images of no pain scenes and ten images of pain scenes adopted from Lei [31] were selected. Two no pain images and two pain images were used in the practice trials. All images were standardized to have identical color contrast, size, and brightness.

#### Target stimuli

Eight fear-related Chinese words (e.g., “处罚” punish in English), eight anger-related words (e.g., “背叛” betray in English), and sixteen positive words (e.g., “盈利” profit in English) for the approach-distancing decision task. One anger-related and one fear-related word and two positive words were used in the practice trials. All words were selected from the Chinese Affective Words System (CAWS) by Wang [38]. The selected words were balanced in terms of valence, arousal, familiarity, specificity, word frequency, total stroke count and separation of emotion dimensions (happiness, sadness, disgust, fear and anger). The first six dimensions were all taken from the CAWS system. In addition, 90 individuals were recruited to evaluate the separated emotion dimension of the selected words (see S3 Table 1).

#### The behavioural activation system (BAS) / behavioural inhibition system (BIS)

Participants’ approach and avoidance tendency were measured by the Chinese version of the BAS/BIS scale [39]. This scale was originally developed by Carver and White [40] and was later translated into Chinese and validated with Chinese participants by Li et al. [39]. The BAS questionnaire, consisting of 13 items, encompasses three subscales: Reward responsiveness (e.g., Upon encountering an appealing opportunity, I promptly experience excitement), Drive (e.g., When I desire something, I typically exert maximal effort to attain it), and Fun seeking (e.g., I frequently act spontaneously without much deliberation). These items measure individuals’ approach motivation and goal-directed behavior in response to rewarding stimuli. The BIS questionnaire, consisting of 7 items (e.g., Even if something bad is about to happen to me, I rarely experience fear or nervousness), which measure individuals’ avoidance motivation and inhibition behaviour in the presence of potential punishment or negative outcomes. Participants had to rate the extent to which the items described themselves on a 4-point scale ranging, with 1 being "completely inconsistent" and 4 being "completely consistent". Higher scores indicate a greater inclination towards approach or avoidance tendencies in individuals.

### Procedure

The participants were tested individually in a quiet room, seated in front of a computer. After giving informed consent, the participant first completed 4 practice trials, followed by 64 experimental trials. For the experimental trials, 8 pain and 8 no pain priming pictures were used. Each priming picture was repeated four times according to word emotion type. For the emotional word, there were three kind words (8 fear-related words, 8 anger-related words, and 16 positive words). Each word was repeated once according to two kinds of priming pictures, resulting in a total of 64 experimental trials.

**Fig. 10:**
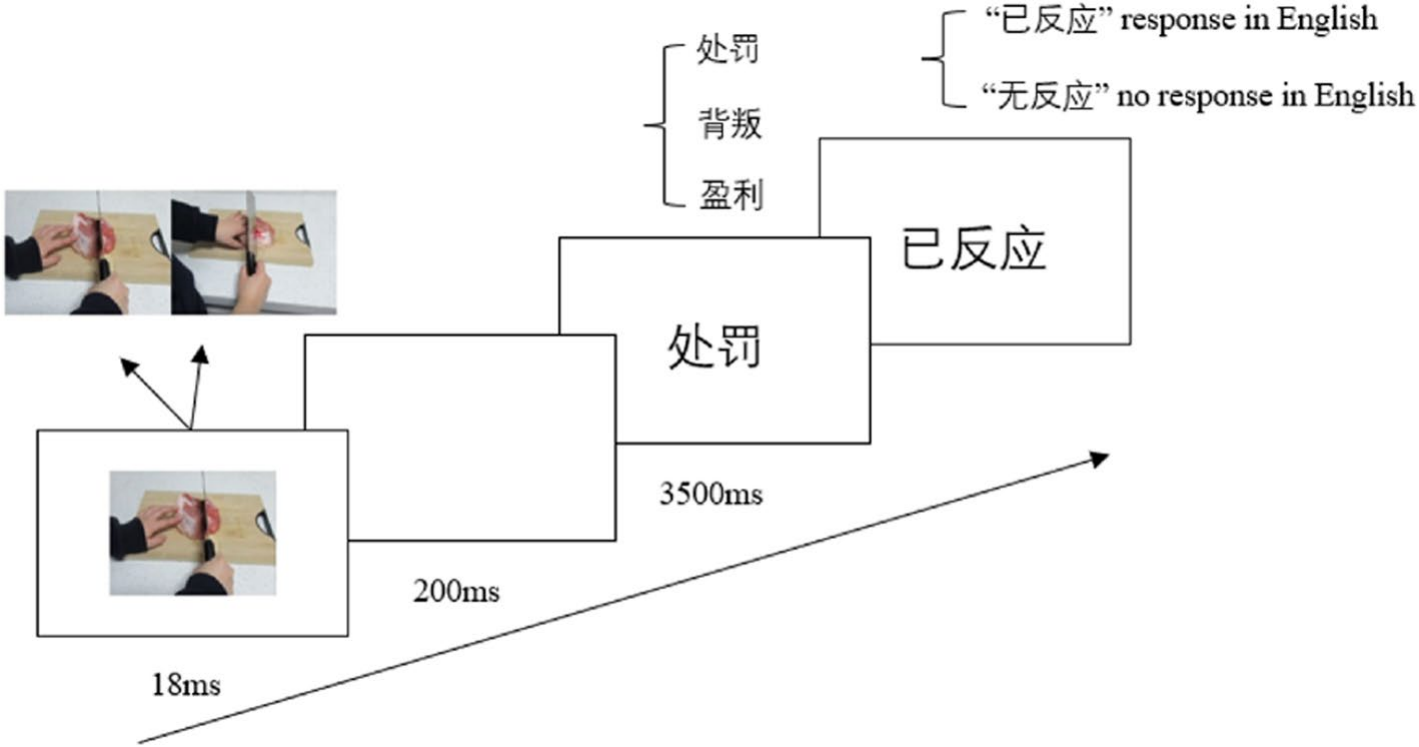
Illustration of the procedure in Experiment 3

Each trial followed the steps outlined in Fig. 10: (1) The priming picture depicting either a pain scene or a no pain scene was displayed for 18ms; (2) A blank screen was shown for 200ms; (3) A two-character Chinese word (fear-related, anger-related or positive) was presented in the centre of the screen. Participants were instructed to decide whether they wanted to approach or avoid that word by pressing the correspondent keys on the keyboard (“F” and “J” keys). Participants had maximally 3500 ms to respond; (4) A feedback screen (response or no response) was displayed for 1500 ms; (5) The inter-trial interval was 1000 ms.

Following the experimental procedure, the participants completed the BAS/BIS questionnaire. Finally, we checked the effectiveness of the subliminal priming by asking participants whether they noticed the flash screen before the word appeared and, if so, whether they were able to identify the content of the flash screen. None of the participants reported perceiving the flash screen. This indicates that the subliminal priming was successful. The entire experiment lasted approximately 10 min.

### Data analysis

Following the exclusion criteria used in Experiment 1, 8.59% of the data was excluded in this experiment.

## Results

### RTs

**Fig. 11:**
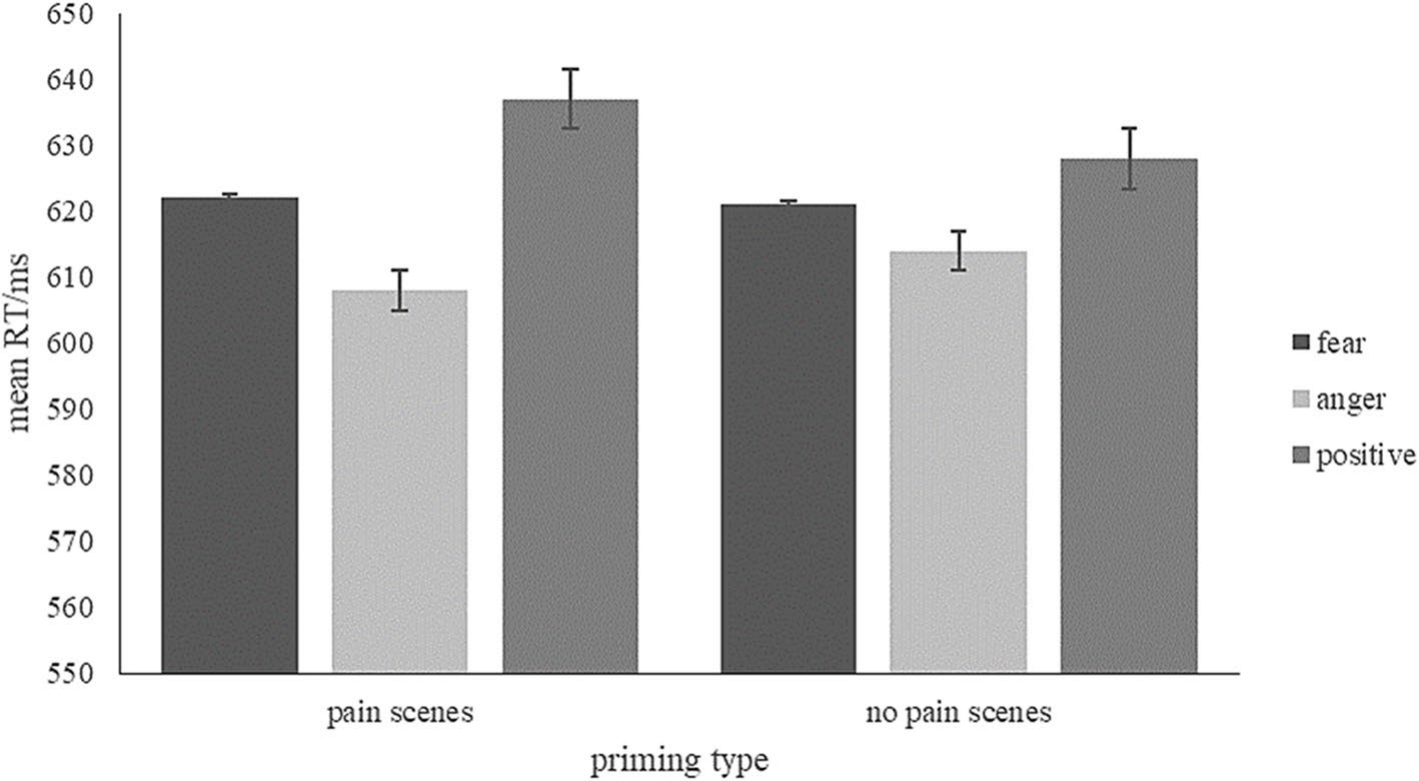
Mean RT of each condition. Error bars represent standard errors

A repeated measure ANCOVA was conducted with the priming type (pain, no pain) and word emotion type (fear-related, anger-related, positive) as the independent variables, the approach and avoidance behaviour tendency scores as the covariable, and the mean RTs of correct responses as the dependent variable. Descriptive statistics are presented in Fig. 11, and full results can be found in S2 Table 2 in the Supplemental Material. The results revealed a significant interaction between the priming type and word emotion type was significant (*F* (2, 76) = 6.96, *p* = 0.002, *η*_*p*_^2^ = 0.16). Paired-sample t-tests revealed that under the pain priming, the RT for anger-related words was significantly shorter compared to positive words (*p* = 0.011), whereas the RT did not differ between fear-related and positive words (*p* = 0.170). Under the no pain priming, RTs did not differ between different emotion word types.

### Accuracy

**Fig. 12:**
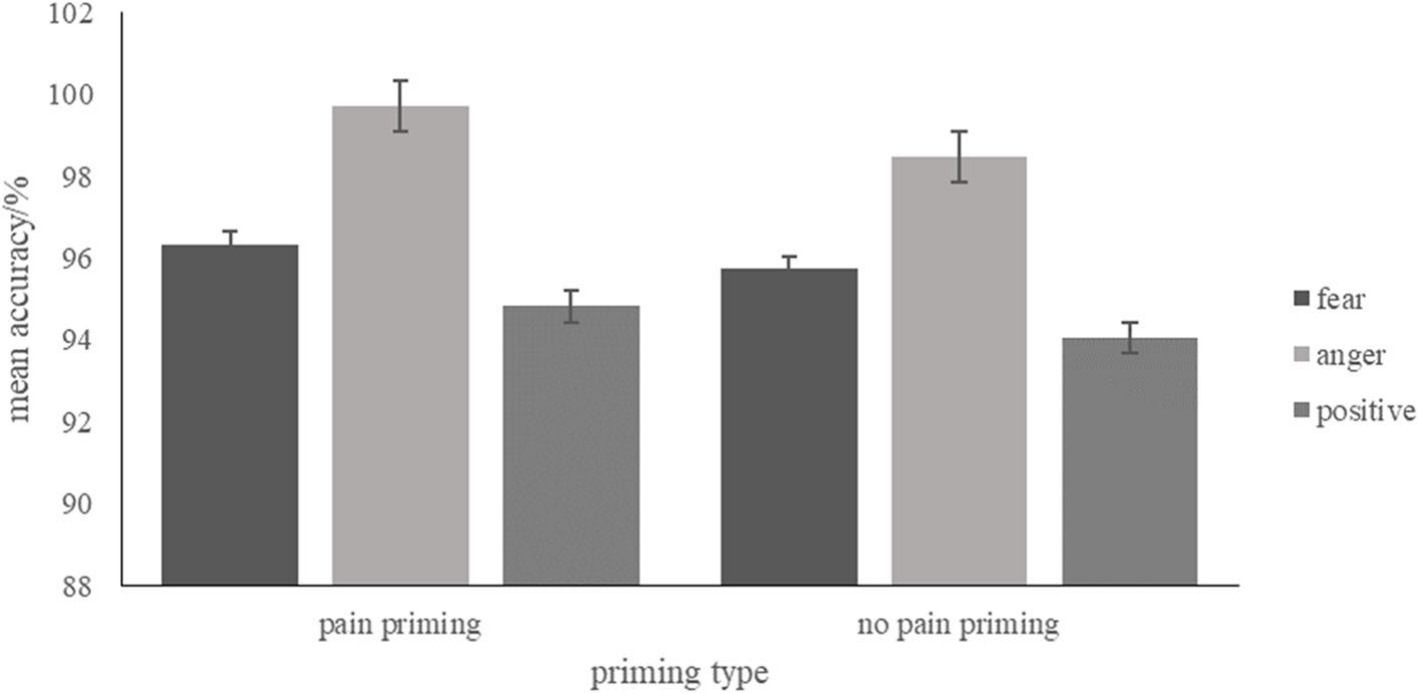
Mean accuracy of each condition. Error bars represent standard errors

A repeated measure ANCOVA was conducted with the priming type (pain, no pain) and word emotion type (fear-related, anger-related, positive) as the independent variables, the approach and avoidance behaviour tendency scores as the covariable, and the proportion of correct responses as the dependent variable as the dependent variable. Approach responses for the positive word and avoidance responses for the negative (fear-related, anger-related) words were counted as correct response. Descriptive statistics are presented in Fig. 12, and full results can be found in S3 Table 3 in the Supplemental Material. Neither the main effects nor the interaction between priming type and the word emotion type were significant.

## Discussion

RT results revealed that, under the pain priming condition, participants exhibited faster avoidance responses to anger-related words compared to approach responses to positive words. However, there was no significant difference in RT between fear-related words and positive words. Previous research suggests that both fear and anger stimuli are perceived as threatening, but they may elicit different behavioral responses. Fear stimuli typically indicate the presence of a potential threat in the environment, prompting individuals to gather more information before taking action. On the other hand, anger stimuli directly pose an immediate threat to individuals, leading to more instantaneous responses [16, 41]. Based on these findings, it can be inferred that anger is more strongly associated with an immediate avoidance response compared to fear in the ADDT. Hence, the RT results suggested that the subliminal primed of the pain scene induced an instantly avoidant response rather than an approach response. Therefore, our findings also supported the TVPH.

The accuracy analyses did not reveal any significant main effects or interaction effects. One possible explanation for this lack of significant findings could be that the participants' accuracy levels had already reached a ceiling level.

## General Discussion

In the present study, three experiments were conducted to test the empathy-altruism hypothesis and TVPH by examining the emotional and behavioural responses after participants perceived subliminal primes of pain and no-pain scenes.

Results from Experiment 1 revealed that subliminal pain perception can lead to a faster response to those negative emotions (i.e., fear) associated with avoidance tendencies than to those negative emotions (i.e., sadness) associated with approaching tendencies. Furthermore, participants exhibited a greater attentional bias towards fearful faces compared to sad faces. This was because individuals directed their attention more rapidly towards fearful faces than towards sad faces when primed with pain. However, the emotional valence of the faces did not influence the difficulty of disengaging attention. Previous research has consistently demonstrated that individuals shifted their attention more quickly from threatening stimuli to neutral ones [42–44]. In the present study, participants exhibited faster attentional shifts under pain priming than under no pain priming, presumably because the pain priming was perceived as a threat. Furthermore, no significant differences were observed in the difficulty of disengaging attention between fearful and sad faces. This finding suggests that both fear and sadness, as negative stimuli, elicit discomfort and increase the likelihood of shifting attention towards neutral faces.

In Experiment 2, we found that participants responded faster to fear-related words than to anger-related words under the pain priming. This suggests that subliminal perception of pain scenes was more strongly related to the negative emotions associated with avoidance tendency (e.g., fear) than to those associated with approaching tendency (e.g., anger). However, the analysis of response accuracy revealed that participants consistently responded more accurately to fear-related words than to anger-related words, regardless of the type of priming employed. The absence of an interaction between priming type and negative word types may be attributed to the relatively simple nature of the lexical decision task used in this experiment, where participants had high accuracy levels, possibly reaching a ceiling effect. Furthermore, the results indicated a consistently higher accuracy for fear-related words compared to anger-related words, regardless of the priming condition. This finding aligns with previous research suggesting that both types of priming stimuli contained elements of danger (e.g., a knife), and dangerous stimuli have been found to be more closely associated with fear rather than anger emotion [45].

In Experiment 3, we found that participants exhibited faster avoidance responses to anger-related words compared to approach responses to positive words during pain priming. This finding suggests that the presentation of pain scenes elicited immediate avoidance responses rather than approach responses. However, it is important to note that the participants' response times for avoidance of fear-related words were not significantly different from their response times for approach towards positive words. This might be because fear stimuli are typically associated with perceived threats in the environment, requiring individuals to engage in cognitive processing before taking action [41]. Similarly, the behavioral tendency represented by positive stimuli contradicts the behavioral tendency induced by pain priming, necessitating individuals to engage in cognitive deliberation before acting. Consequently, no significant difference in response times was observed between fear-related words and positive words. Furthermore, we did not observe any significant main effects or interactions for in our accuracy analyses. This might be because participants’ accuracy has reached a ceiling level.

The results from the three experiments consistently demonstrated a relationship between physical pain scenes, fear emotions, and avoidance tendencies. These findings suggest that when individuals observe others experiencing physical pain, it triggers feelings of fear and prompts avoidance responses. These results provide support for the TVPH, which posits that individuals are more likely to respond with avoidance behaviors when faced with potential physical harm.

The results supporting the TVPH over the empathy-altruism hypothesis may be attributed to two possible reasons. Firstly, the duration of presentation for the pain scenes could play a role. Studies suggest that pain signals undergo distinct processing stages [46]. Goubert et al. [47] proposed that pain-related signals automatically activate the observer's threat system, inducing self-directed emotions like personal distress. Subsequent stages involve the interpretation of pain cues influenced by the observer's background, potentially leading to other-directed emotions like empathic concern. Studies supporting the empathy-altruism hypothesis often employ longer presentations of protagonists' painful experiences through stories (e.g., 40–60 s) [7, 8]. In contrast, studies supporting the TVPH use shorter presentations of pain stimuli, such as pictures or words (e.g., 13.3–200 ms) [1, 4, 5]. In our study, subliminal pain scene priming lasted only 18 ms, and therefore our findings were aligning with the TVPH. Subliminal stimuli as priming may yield more reliable and practical findings, as our daily information processing primarily occurs unconsciously. Additionally, subliminal stimuli directly process through subcortical pathways and resist conscious control [48, 49], potentially reducing susceptibility to external stimuli and personal experiences. Secondly, the type of observed pain may contribute. Previous research indicates that witnessing physical pain evokes personal distress, whereas observing social pain elicits empathic concern [7, 9]. Our study employed pictures depicting physical pain as the priming stimulus, thus supporting the TVPH.

Our findings contribute to previous research in several key areas. Firstly, prior studies have established a strong association between heightened pain responses and the priming of high threat value stimuli [49–51]. However, our study directly examined the impact of pain perception on participants' emotional and behavioral responses. Across all three experiments, we consistently observed that physical pain perception resulted in an increased attentional bias towards fear emotions and faster avoidance responses. Secondly, the attentional bias index analyses conducted in Experiment 1 provided valuable insights into the origins of the attentional bias towards fear emotion. The results indicated that the observed bias was primarily driven by faster attentional orientation towards fear emotion, rather than slower disengagement from other emotions. Lastly, while previous research [10] has demonstrated that pain perception elicits faster avoidance responses and slower approach responses, inferred from participants' button release and button press speed in a go/no go task. Experiment 3 further elucidated that pain perception led to explicit faster avoidance responses towards negative emotions and slower approach responses towards positive emotions. Thus, our findings expand upon existing knowledge and shed light on the attentional biases, emotional responses, and approach-avoidance tendencies associated with pain perception.

It is worth noting that, following pain scene priming, participants responded faster to fear-related words than to anger-related words in the lexical decision task of Experiment 2. However, they responded equally fast to these two types of words in the approach/distancing decision task of Experiment 3. This might be because the subliminal perception of others’ pain induced an immediate avoidance response, which was reflected by the faster response to the fear-related words in the LDT, where participants' implicit approach-avoidance response was measured. In contrast, in the ADDT, participants' approach-avoidance response was measured explicitly. Therefore, although the subliminal perception of others’ pains still reduced the reaction time to the fear-related words, this effect was cancelled by the factor that people generally respond faster to anger-related words in ADDT [37].

The study has some potential limitations that warrant further exploration. Firstly, all priming stimuli used in this study were picture stimuli, and it remains uncertain whether the same findings would be observed with word stimuli as priming stimuli. Sun et al. [29] suggested that pictures may have a more direct emotional impact than words. Future research could investigate the effects of subliminal pain word priming on participants' emotional and behavioral responses. Secondly, the present study exclusively focused on the effect of physical pain perception on emotional and behavioural responses, leaving the effect of social pain perception unclear. As mentioned by [52], perception of physical pain is likely to rely on automatic, low-level processes activated through bottom-up mechanisms, while social pain perception may demand more deliberate effort and high-level processing, such as understanding the other person's mental state. Future studies should compare the effects of physical and social pain perception on emotional and behavioral responses, ideally using a within-subject design. Lastly, future research could utilize experimental tools with a higher temporal resolution, such as eye-movement tracking technology and event-related potentials, to gain deeper insights into the temporal course of the related effects of pain scene perception. For instance, as proposed by Yan et al. [46], pain-related signals might automatically activate the observer's threat system and self-directed emotions (e.g., personal distress) in the early phase. In later phases, the interpretation of pain cues might be influenced by the observer's background and characteristics, leading to other-directed emotions (e.g., empathic concern).

## Conclusion

In summary, our study demonstrated that the subliminal perception of others’ physical pain led to faster response and attentional bias to fearful faces than sad faces (Experiment 1), faster responses to fear-related words than to anger-related words (Experiment 2), and faster avoidance response to anger-related words than approach response to positive words (Experiment 3). These findings indicate a strong association between perceiving others' pain, fear emotion, and immediate avoidance responses. Our study's results provide supporting evidence for the TVPH.

### Supplementary Information

**Additional file 1:** **S1 Table 1.** The results of RT in Experiment 1. **S1 Table 2.** The results of RT in Experiment 1. **S1 Table 3.** The results of RT in Experiment 1. **S1 Table 4.** The results of attentional bias score in Experiment 1. **S1 Table 5.** The results of attentional orientation acceleration score in Experiment 1. **S1 Table 6.** The results of attentional disengagement difficulty score in Experiment 1. **S2 Table 1.** The score of each dimension of target stimulus in Experiment 2. **S2 Table2.** The results of RT in Experiment 2. **S2 Table3.** The results of accuracy in Experiment 2. **S3 Table 1.** The score of each dimension of target stimulus in Experiment 3. **S3 Table2.** The results of RT in Experiment 3. **S3 Table3.** The results of accuracy in Experiment 3.

## Data Availability

All materials in the manuscript, including all relevant raw data, can freely be made available to any person wishing to use them for non-commercial purposes, without breaching participant confidentiality. The datasets generated and/or analyzed in the present study are available from the corresponding author. Data collection and analysis were performed under the supervision of the corresponding author.

## Abbreviations

ADDT: Approach-Distancing Decision Task
BAS: Behavioural Inhibition System
BIS: Behavioural Activation System
CAWS: Chinese Affective Words System
LDT: Lexical Decision Task
TVPH: Threat Value of Pain Hypothesis
VDT: Valence Decision Task
