# Supplementary material for: Subliminal perception of others’ physical pain induces personal distress rather than empathic concern
Source: BMC Psychol. 2023 Sep 15;11:276. doi: 10.1186/s40359-023-01310-3 (PMC10503136; doi:10.1186/s40359-023-01310-3)
Supplement: Supplementary file 1 — Additional file 1: S1 Table 1. The results of RT in Experiment 1. S1 Table 2. The results of RT in Experiment 1. S1 Table 3. The results of RT in Experiment 1. S1 Table 4. The results of attentional bias score in Experiment 1. S1 Table 5. The results of attentional orientation acceleration score in Experiment 1. S1 Table 6. The results of attentional disengagement difficulty score in Experiment 1. S2 Table 1. The score of each dimension of target stimulus in Experiment 2. S2 Table2. The results of RT in Experiment 2. S2 Table3. The results of accuracy in Experiment 2. S3 Table 1. The score of each dimension of target stimulus in Experiment 3. S3 Table2. The results of RT in Experiment 3. S3 Table3. The results of accuracy in Experiment 3. [file 40359_2023_1310_MOESM1_ESM.docx]

**S1 Table1.** The results of RT in Experiment 1

|  | ***df*** | ***F*** | ***p*** | **partial *η^2^*** | |
| --- | --- | --- | --- | --- | --- |
| priming type | (1, 24) | 0.92 | .347 | | 0.04 |
| negative emotion type | (1, 24) | 4.94 | **.036** | | 0.17 |
| the site of probe dot | (1, 24) | 12.04 | **.002** | | 0.33 |
| priming type × negative emotion type | (1, 24) | 14.77 | **.001** | | 0.38 |
| priming type × the site of probe dot | (1, 24) | 12.40 | **.002** | | 0.34 |
| negative emotion type × the site of probe dot | (1, 24) | 0.229 | .636 | | 0.01 |
| priming type × negative emotion type × the site of probe dot | (1, 24) | 5.71 | **.025** | | 0.19 |

Note. Bold means *p* < .050. The same below.

**S1 Table2.** The results of RT in Experiment 1

|  | ***df*** | ***F*** | ***p*** | **partial *η^2^*** | |
| --- | --- | --- | --- | --- | --- |
| negative emotion type | (1, 24) | 0.15 | .704 | | 0.01 |
| the site of probe dot | (1, 24) | 21.45 | **<.001** | | 0.47 |
| negative emotion type × the site of probe dot | (1, 24) | 5.37 | **.029** | | 0.18 |

**S1 Table3.** The results of RT in Experiment 1

|  | ***df*** | ***F*** | ***p*** | **partial *η^2^*** | |
| --- | --- | --- | --- | --- | --- |
| negative emotion type | (1, 24) | 25.48 | **<.001** | | .52 |
| the site of probe dot | (1, 24) | 0.45 | .51 | | .02 |
| negative emotion type × the site of probe dot | (1, 24) | 1.24 | .28 | | .05 |

**S1 Table4.** The results of attentional bias score in Experiment 1

|  | ***df*** | ***F*** | ***p*** | **partial *η^2^*** | |
| --- | --- | --- | --- | --- | --- |
| priming type | (1, 24) | 12.40 | **.002** | | .34 |
| negative emotion type | (1, 24) | 0.23 | .636 | | .01 |
| priming type × negative emotion type | (1, 24) | 5.71 | **.025** | | .19 |

**S1 Table5.** The results of attentional orientation acceleration score in Experiment 1

|  | ***df*** | ***F*** | ***p*** | | **partial *η^2^*** | |
| --- | --- | --- | --- | --- | --- | --- |
| priming type | (1, 24) | 0.74 | | .400 | | .03 |
| negative emotion type | (1, 24) | 1.25 | | .275 | | .05 |
| priming type × negative emotion type | (1, 24) | 20.49 | | **<.001** | | .46 |

**S1 Table6.** The results of attentional disengagement difficulty score in Experiment 1

|  | ***df*** | ***F*** | ***p*** | **partial *η^2^*** | |
| --- | --- | --- | --- | --- | --- |
| priming type | (1, 24) | 8.63 | **.007** | | .26 |
| negative emotion type | (1, 24) | 4.53 | **.044** | | .16 |
| priming type × negative emotion type | (1, 24) | 0.21 | .655 | | .01 |

**S2 Table 1.** The score of each dimension of target stimulus in Experiment 2

|  | **fear words（N=9）** | **anger words（N=9）** | **neutral words（N=9）** |
| --- | --- | --- | --- |
| valence | 3.16（0.22） | 3.06（0.30） | 5.10（0.68） |
| arousal | 5.34（0.85） | 5.58（0.39） | 3.99（0.48） |
| familiarity | 4.85（0.50） | 4.75（0.31） | 5.01（0.48） |
| concreteness | 3.51（0.55） | 4.22（0.88） | 3.55（1.23） |
| frequency | 40.11（50.43） | 12.56（9.15） | 33.56（45.70） |
| number of strokes | 18.78（5.14） | 22.33（5.66） | 18.00（2.50） |
| happiness | 1.34（0.18） | 1.35（0.22） | 2.47（0.28） |
| sadness | 3.22（0.28） | 2.99（0.46） | 1.96（0.29） |
| fear | 3.88（0.38） | 2.91（0.37） | 1.97（0.24） |
| anger | 2.99（0.45） | 3.82（0.48） | 1.83（0.31） |
| disgust | 3.22（0.36） | 3.58（0.48） | 1.82（0.25） |

**S2 Table2.** The results of RT in Experiment 2

|  | ***df*** | ***F*** | ***p*** | **partial *η^2^*** | |
| --- | --- | --- | --- | --- | --- |
| priming type | (1, 24) | 0.06 | .805 | | < .01 |
| word emotion type | (2, 48) | 7.62 | **.003** | | .24 |
| priming type × word emotion type | (2, 48) | 3.47 | **.039** | | .13 |

**S2 Table3.** The results of accuracy in Experiment 2

|  | ***df*** | ***F*** | ***p*** | **partial *η^2^*** | |
| --- | --- | --- | --- | --- | --- |
| priming type | (1, 24) | 0.14 | 0.714 | | .01 |
| word emotion type | (2, 48) | 7.28 | **.004** | | .23 |
| priming type × word emotion type | (2, 48) | 0.10 | .908 | | < .01 |

**S3 Table 1.** The score of each dimension of target stimulus in Experiment 3

|  | **fear words（N=8）** | **anger words（N=8）** | **positive words（N=8）** |
| --- | --- | --- | --- |
| valence | 3.22（0.40） | 3.05（0.21） | 6.66（0.34） |
| arousal | 5.53（0.72） | 5.69（0.52） | 5.28（0.50） |
| familiarity | 5.09（0.30） | 5.02（0.43） | 5.14（0.29） |
| concreteness | 3.63（0.71） | 3.71（0.99） | 3.92（0.75） |
| frequency | 40.13（55.11） | 146.25（376.62） | 12.00（9.95） |
| number of strokes | 16.25（5.26） | 19.38（6.63） | 15.19（4.02） |
| happiness | 1.40（0.22） | 1.34（0.29） | 3.91（0.44） |
| sadness | 3.25（0.42） | 2.87（0.46） | 1.59（0.32） |
| fear | 3.74（0.52） | 2.81（0.34） | 1.64（0.28） |
| anger | 3.00（0.48） | 3.86（0.49） | 1.47（0.26） |
| disgust | 3.19（0.38） | 3.56（0.39） | 1.52（0.28） |

**S3 Table2.** The results of RT in Experiment 3

|  | ***df*** | ***F*** | ***p*** | **partial *η^2^*** | |
| --- | --- | --- | --- | --- | --- |
| priming type | (1, 38) | 0.00 | .955 | | <.01 |
| word emotion type | (2, 76) | 0.59 | .557 | | .02 |
| priming type × word emotion type | (2, 76) | 6.96 | **.002** | | .16 |

**S3 Table3.** The results of accuracy in Experiment 3

|  | ***df*** | ***F*** | ***p*** | **partial *η^2^*** | |
| --- | --- | --- | --- | --- | --- |
| priming type | (1, 38) | 0.28 | .601 | | .01 |
| word emotion type | (2, 76) | 0.91 | .384 | | .02 |
| priming type × word emotion type | (2, 76) | 0.44 | .610 | | .01 |
